# Evaluation of Eco-Efficient Concretes Produced with Fly Ash and Uncarbonated Recycled Aggregates

**DOI:** 10.3390/ma14247499

**Published:** 2021-12-07

**Authors:** Miren Etxeberria

**Affiliations:** Department of Civil and Environmental Engineering, Universitat Politècnica de Catalunya·BarcelonaTECH, Campus Nord, 08034 Barcelona, Spain; miren.etxeberria@upc.edu

**Keywords:** eco-efficiency, recycled aggregate concrete, fly ash, mechanical properties, carbonation, chloride resistance, durability, sustainable construction

## Abstract

The fabrication of conventional concrete, as well as remains from demolition, has a high environmental impact. This paper assessed the eco-efficiency of concrete made with uncarbonated recycled concrete aggregates (RCA) and fly ash (FA). Two concrete series were produced with an effective water/cement ratio of 0.50 (Series 1) and 0.40 (Series 2). In both series, concretes were produced using 0% and 50% of RCA with 0%, 25% and 50% FA. After analysing the compressive strength, and carbonation and chloride resistance of those concretes, their eco-efficiency based on the binder intensity and CO_2_-eq intensity was assessed. We found that the use of 50% uncarbonated RCA improved the properties of concretes produced with FA with respect to using natural aggregates. The concrete made of 25% FA plus RCA was considered the most eco-efficient based on the tests of compressive, carbonation and chloride properties with the values of 4.1 kg CO_2_ m^−3^ MPa^−1^, 76.3 kg CO_2_ m^−3^ mm^−1^ year^0.5^ and 0.079 kg CO_2_ m^−3^ C^−1^, respectively. The uncarbonated RCA improved carbonation resistance, and FA improved chloride resistance. It can be concluded that the use of 50% un-carbonated RCA combined with FA considerably enhanced the properties of hardened concrete and their eco-efficiency with respect to concretes produced with natural aggregates.

## 1. Introduction

Concrete production has a high environmental impact due to the abundant energy consumption and CO_2_ emissions of cement production, releasing approximately 5–7% of total global anthropogenic CO_2_ emissions and 3% of total greenhouse gases emissions in the atmosphere [1,2,3]. In general, the impact of concrete is assessed as a functional unit of the amount of cement employed in the concrete on the environment weight or volume [4]. Adequate performance in terms of mechanical and durability properties guarantees the eco-efficiency of concrete [4]. It can be said that the demolition of concrete structures causes a considerable volume of waste that terminates in landfills. In 2018, the construction and demolition waste was 35.4% of the total waste (2277 million tonnes) generated in the EU by all economic activities and households, of which only 54.2% was recovered [5,6]. In this vein, the use of supplementary cementitious materials, such as fly ash (FA) and recycled concrete aggregates (RCA, recycled aggregates produced crushing concrete structures), in the production of green concrete (CRAFA concrete) can help increase the eco-efficiency of concretes [7,8,9,10].

RCA has been proven to be suitable for concrete production [11]. In addition, the employment of recycled aggregates concrete (RAC, concrete produced using recycled aggregates) as a structural material has been widely analysed and validated in many applications [12,13]. However, taking into consideration its durability properties, it was found that the RAC concrete had lower chloride ion diffusion resistance than natural aggregate concrete (NAC) when concretes were produced with ordinary Portland cement (OPC) [14,15,16,17]. The studies on carbonation resistance tend to be inconsistent. Guo et al. [15], Adessina et al. [17] and Silva et al. [18], among other researchers, stated that RAC produced with a high percentage of RCA achieved higher carbonation depth than NAC. Zeng [19] stated that the best replacement percentage of natural aggregates with RCA was 50% as this avoided a decrease in carbonation resistance.

However, certain researchers [16,20] have stated that the RAC mixtures had similar or higher resistance to carbonation than NAC due to the old adhered mortar. According to Leemann and Loser [21], there was no systematic difference in the carbonation coefficient independently of the replacement levels of RCA. Although the RCA particles that are either porous or already carbonated at the time of concrete production can lead to a local increase of carbonation depth, the impact on the carbonation coefficient is not significant, as there is only an increase of 10% at a given compressive strength [21]. Pedro et al. [22] documented that when the quality of the cement paste in the new concrete was found to be considerably better than that of the RCA, an increase of the carbonation coefficient with increasing levels of NAC replacement was inevitable.

Today, the use of FA in proportions of up to 15–30% [23,24] in concrete production is usual. The use of FA causes low strength in concretes at initial ages [25]. However, at later ages, the strength and durability of concrete increases [24,26,27]. In addition, the chloride ion penetration resistance of concrete is higher when FA is employed [28,29]. However, the concrete produced with FA achieves lower carbonation resistance than the concrete made of 100% OPC [30].

The concrete produced with a combination of FA and RCA (CRAFA) could improve durability and eco-efficiency required for new sustainable concretes [31]. According to Faella et al. [32] and Kurda et al. [33], the CRAFA concrete (produced with 50% RCA and 50% FA) exhibited lower water permeability compared with NAC concrete after 28 days, achieving a similar compressive strength with the NAC (produced with OPC) at 90 days. Moreover, a significant attenuation of the chloride-ion penetration was achieved by adding FA to the RAC mixtures [32]. Kurda et al. [33], Kim et al. [34] and Kou and Poon [35], among other researchers, determined that the total charge passed through concrete (measured in C) increased with increasing levels of coarse RCA replacement, and the opposite occurred when increasing the incorporation levels of FA. According to Corinaldesi and Moriconi [36], the addition of FA proved to be effective in reducing the chloride ion penetration depth in concrete, even when RCA was used. Moreover, they concluded that the addition of FA to RAC increased the carbonation rate. Similar results were obtained by Faella et al. [32], Jianmin et al. [37] and Poon and Kou [35,38].

However, the CRAFA mixtures were prepared with a lower water/binder ratio [36]; as a result of the refinement of the pore system, carbonation did not present risks of reinforcement corrosion. Moreover, Tian et al. [39] concluded that the influence that had the most impact on the depth of carbonation of the CRAFA was that of the water/binder ratio, signifying that the carbonation age and the FA content had a minor effect. Xiao et al. [20] found that although the use of FA in RAC production improved the internal pore structure by decreasing the porosity of the concrete, it also resulted in the reduction of the total alkaline content that can be carbonated, resulting in greater carbonation depths.

The eco-efficiency of the cement used in concrete production was first analysed by Damineli et al. [4]. The optimum mix design is calculated by determining the binder intensity (Bi) and CO_2_-eq intensity (Ci) of the concrete. The Bi value measures the amount of binder necessary to deliver a unit of strength and, consequently, the efficiency of the employed binder. It has been suggested that the minimum stabilised Bi value at 28 days after production is 5 kg m^−3^ MPa^−1^ for concretes of >60 MPa. Research suggests that this value will be an achievable goal for normal strength concrete (<50 MPa) in the near future and the ready-mix concretes <40 MPa consume the most cement, having a Bi value of 10–12 kg m^−3^ MPa^−1^. For 20 MPa concretes, the minimum Bi value is approximately 13 kg m^−3^ MPa^−1^ [4]. A higher Bi value (>20 kg m^−3^ MPa^−1^) is necessary when large volumes of weak or low elastic modulus waste materials are employed in concrete production. To achieve a moderate Bi value, needed for higher concrete strengths, the binder consumption per unit of MPa should always be low [40].

The Ci value of concretes is determined by dividing the emitted CO_2_-eq in kg per m^3^ of concrete by its compressive strength at 28 days, and it is expressed as kg CO_2_ m^−3^ MPa^−1^. The Ci value considers the contribution of concrete mixtures to the global warming potential (GWP) per unit volume and strength, and it has been analysed intensively during recent years [3,41,42]. Damineli et al. [4], studying international data, determined that the average estimated Ci value was 7.1 kg CO_2_ m^−3^ MPa^−1^. All Ci values > 13 kg CO_2_ m^−3^ MPa^−1^ presented a high Bi value and no clinker replacement [4]. The data suggest [4] that the mix design and the selection of raw materials allow reducing Ci values by a factor of 10, from 15 kg CO_2_ m^−3^ MPa^−1^ to a minimum of 1.5 kg CO_2_ m^−3^ MPa^−1^. The minimum estimated Ci value was 1.5–2 kg CO_2_ m^−3^ MPa^−1^. According to de Matos et al. [41], all concretes with Ci < 5.0 kg CO_2_ m^−3^ MPa^−1^ are in the range of 70–80 MPa, while for the range of 50–60 MPa, the average Ci value is 6.2 kg CO_2_ m^−3^ MPa^−1^. Similar values have been obtained by Celic et al. [43]. Randl et al. [42] also confirmed that the use of FA and the improved mix design were an effective solution for reducing Ci values of concretes.

This paper analyses the beneficial application of 50% of uncarbonated RCA as a substitution for natural aggregates together with 0%, 25% and 50% of FA in the replacement of cement for adequate properties of CRAFA concrete production. The compressive strength and durability properties of carbonation and chloride resistance were determined and compared with conventional concrete. The eco-efficiency of the produced concretes was assessed by measuring Bi and Ci values with respect to those obtained for compressive strength, carbonation coefficient and chloride resistance. The results guaranteed that CRAFA concrete (produced with FA and RCA) could maintain high strength and durability properties. Consequently, CRAFA achieved a higher level of eco-efficiency than the concretes made with Portland cement and natural aggregates.

## 2. Materials and Methods

### 2.1. Binder

The CEM II A-L 42.5R cement (88% clinker, 10% limestone and 2 minority component; CEM II) was employed in the concrete production. Class F coal-fly ash was used at 0%, 25% and 50% to replace CEM II. The CEM I 52.5R cement (high strength and rapidly hardened, 98% clinker and 2% of minority component; CEM I) was used, replaced by FA by 50%. CEM II and CEM I were produced by the same cement producer using the same type of clinker for their manufacture. The density of both types of cement was 3.1 kg/dm^3^. The coal-FA produced by a Spanish coal-fired power plant had a density of 2.16 kg/dm^3^. Table 1 shows the composition of the binder materials.

#### Aggregates

**Figure 1:**
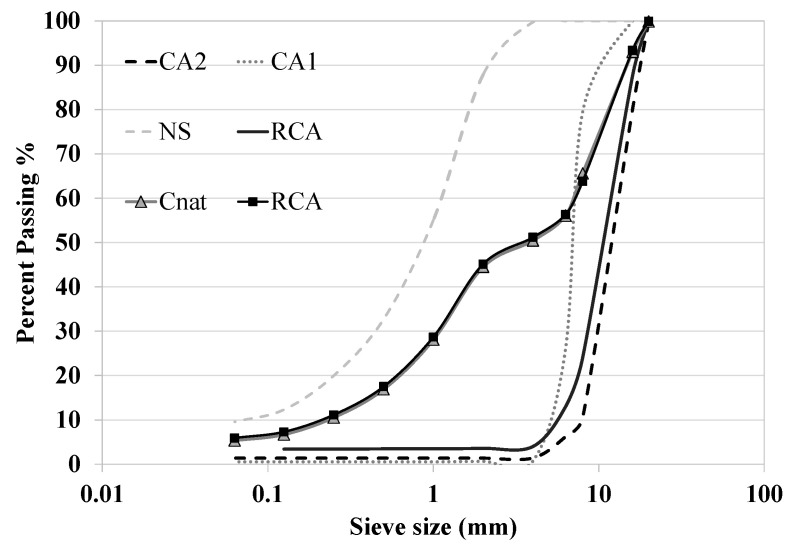
The grading distribution of all the aggregate fractions (NS, CA1, CA2 and RCA) and the corresponding mixtures used for concrete production with 100% of natural aggregates (C) and 50% coarse recycled concrete aggregates (RCA).

Three fractions of natural limestone aggregates were used to produce concrete: fine natural aggregates (NS of 0/4 mm) and two natural coarse aggregates (CA1 of 5/10 mm and CA2 of 8/20 mm). The grading distribution of the aggregates (Figure 1) was determined following EN 933-1 specification. The dry density of NS, CA1 and CA2 was 2.59 kg/dm^3^, 2.64 kg/dm^3^ and 2.66 kg/dm^3^, respectively. The absorption capacity was 1.79%, 0.69% and 0.57%, respectively. The dry density and absorption capacity were determined following the EN 1097-6 specifications.

**Figure 2:**
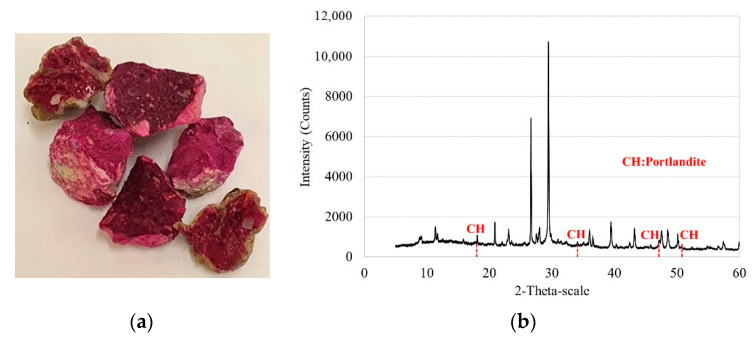
Recycled aggregates were uncarbonated: (**a**) the phenolphthalein (pink colour) showed the presence of Ca(OH)_2_; (**b**) DRX analysis shows Ca(OH)_2_.

The RCA aggregates were produced by crushing a one-year-old parent concrete, specifically, 30 MPa cylindrical concrete samples (150 mm Ø and 300 mm long) obtained from a concrete manufacturing company. The component of the parent concrete was CEM II A-L 42.5R cement and limestone aggregates. RCA was used for concrete production immediately after the parent concrete was crushed. The parent concrete presented a carbonation depth of 3–4 mm. In consequence, the RCA aggregates were uncarbonated. Figure 2 illustrates that the RCA was uncarbonated: (a) pink colour by phenolphthalein; (b) DRX analysis, in which Ca(OH)_2_ component is shown. The grading distribution of RCA was a 5/20 mm fraction (Figure 1). Its dry density was 2.33 kg/dm^3^ and absorption capacity 5.35%. The RCA had a lower density and higher absorption than that of the limestone aggregates (calcium carbonate).

### 2.2. Mix Design and Production Process

Two series of concrete production were carried out, employing an effective water/cement ratio of 0.50 for Series 1 and 0.40 for Series 2 (Table 2). Each concrete was assigned the numeration of −1 or −2 depending on the manufacturing series 1 or 2, respectively.

**Figure 3:**
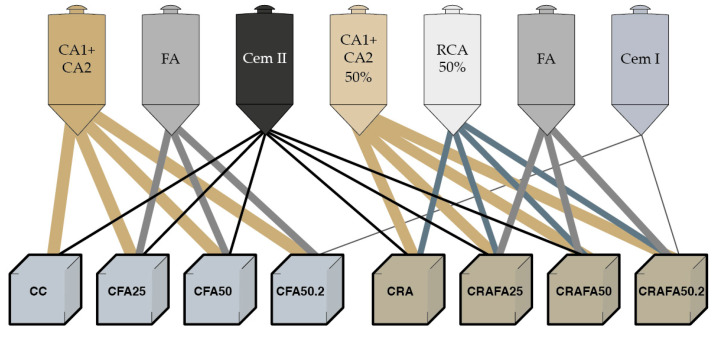
Description of materials employed and produced concrete. The eight concretes were produced in both series.

In each series, eight different concretes were produced. Four types of binders were employed: (1) 100% CEM II cement; (2) 75% CEM II cement plus 25% FA (FA25); (3) 50% CEM II cement plus 50% FA (FA50); and (4) 50% CEM I cement plus 50% of FA (FA50.2). Those binders were mixed with two types of aggregate fractions: (1) 100% of natural aggregates and (2) 50% of RCA in replacement (in volume) of natural coarse aggregates. All the concretes were produced employing natural sand. The grading distribution of the different aggregate fraction mixtures is described in Figure 2. The concrete produced with 100% of natural aggregates and the four binders previously mentioned were designated as CC, CFA25, CFA50 and CFA50.2, respectively. The assignation of concretes produced with 50% of RCA and the same four binders were CRA, CRAFA25, CRAFA50 and CRAFA50.2. Figure 3 shows the manufactured concrete and the employed materials.

Following Neville’s [44] definition of the effective amount of water in the mixture, which occupies the space outside the aggregate particles, the effective water/cement ratio for Series 1 (0.50) and Series 2 (0.40) was kept constant. The reason for this was to achieve the same conditions with respect to the hydration of the cement paste caused by the high absorption of RCA. RCA was used with high moisture content in nearly saturated surface-dry conditions (80–90% of water absorption capacity), but not totally saturated in order to avoid the influence of water surface layers on the mechanical properties of the concrete [45]. The RCA moisture content was measured before its use and the dosages were adjusted according to the remaining effective water absorption capacity (the effective water absorption of the aggregates was determined by submerging them in water for 20 min) of RCA and the natural aggregates. While the effective absorption capacity of NS was 90% and that of CA1 and CA2 50% of their total absorption capacity, the effective absorption capacity of RCA was 70%.

A superplasticiser based on modified polycarboxylates was employed in all mixtures, at 0.50% with respect to the cement’s weight in all the concrete mixtures produced in Series 1 (Table 2) to achieve a slump of 190–200 mm. In Series 2, the superplasticiser dosage was increased to 1–1.6% to achieve a slump test of 160–210 mm. The slump value was determined following the UNE-EN 12350-2:2020 specifications [46].

The concrete samples were produced and cured following UNE-EN 12390-2:2001 regulations [47] and manually compacted using a steel rod. The concrete samples were then covered with a plastic sheet and air-cured for the first 24 h. After 24 h of casting, the samples were demoulded and then stored at a temperature of 21 °C and 95% humidity until reaching the test ages for the physical, mechanical and chloride ion penetration tests. The samples that were submitted to an accelerated carbonation process were taken from the humidity room after 28 days and placed in the laboratory under pre-conditioning for 14 days at a CO_2_ concentration of 400 ppm, 21 ± 1 °C and 50–55% humidity before then being placed in the CO_2_ chamber.

### 2.3. Test Procedure

#### 2.3.1. Compressive Strength

The compressive strength of concretes was determined using a compression machine with a loading capacity of 3000 kN. The compressive strength was measured at the ages of 7, 28 and 90 days following the EN 12390-3:2009 specifications [48]. Three cubic samples (100 × 100 × 100 mm) were used for each concrete mixture, at a total of nine samples.

#### 2.3.2. Carbonation and Chloride Resistance

Concretes’ carbonation resistance was determined by analysing the carbonation depth presented by concrete specimens after being submitted to accelerated carbonation for 0, 7 and 14 days. Six cylindrical samples of 100 mm Ø and 100 mm long and six cubic samples of 100 × 100 × 100 mm were analysed for each concrete. All the samples were cured in the humidity room for 28 days and were later exposed to a pre-conditioning period of 2 weeks under laboratory conditions (21 ± 1 °C and 50–55% humidity) before their exposure to accelerated carbonation. After the pre-conditioning period (0 day), the carbonation depth and compressive strength of the first concrete samples, two cylindrical samples of 100 mm Ø and 100 mm long and two cubic samples of 100 × 100 × 100 mm of each concrete mixture were tested, respectively. The carbonation depth was determined by spraying a colour indicator solution on a freshly broken surface. A solution composed of 1 g of phenolphthalein indicator in 70 mL of ethanol and 30 mL of demineralised water was applied following EN specifications. The four cylindrical samples of 100 mm Ø and 100 mm long and four cubic samples (100 × 100 × 100 mm) of each concrete mixture were placed in a climate chamber with CO_2_ control for the accelerated carbonation test at 20% CO_2_, 20 °C and 60% humidity, following GB T50082-2009 standards [49]. After 7 and 14 days of exposure, two cylindrical samples for each concrete mixture were measured for carbonation depth. Additionally, the compressive strength of the cubic samples submitted to carbonation was determined.

The 20% CO_2_ concentration was found to be excessive. It is known that the carbonation coefficient increases with increased CO_2_ concentration [50]. Moreover, at long test periods, the high concentrations of CO_2_ cause modifications in the microstructure of the cement paste when compared to samples subjected to natural exposure only [51]. However, subjecting the concrete test samples to conditions of high concentration of CO_2_ over short periods of testing (for 14 days in this study) allows for the determining of the concrete’s resistance to carbonation [52].

The chloride penetrability of the concrete was determined following the American Standard Test Method, ASTM C1202 standards [53], using a 50 mm thick and 100 mm Ø concrete disc cut from the 100 mm Ø and 200 mm length concrete cylinder. The concrete’s resistance to chloride-ion penetration is represented by the total charge (measured in C) during a test period of 6 h, maintaining a potential difference of 60 V dc across both ends of the specimens. In this study, the chloride ion penetrability test was carried out on the concrete samples at 28 and 90 days. Each result was the average of three measurements.

#### 2.3.3. Eco-Efficiency Assessment

Bi and Ci values were used to assess the concretes’ eco-efficiency as defined by Damineli et al. [4]. Three Bi values, the weight of binder (kg) employed for one m^3^ of concrete production to deliver one unit of performance, compressive strength (Bi-comp), performance of carbonation coefficient (Bi-carb) and chloride resistance (Bi-chlor), were determined for each concrete. For each type of concrete, three Ci values were also determined, as the amount of CO_2_ emitted per unit of compressive strength (Ci-comp), per carbonation coefficient (Ci-carb) and per chloride resistance (Ci-chlor).

The CO_2_ emissions (kg) per weight of CEM II and CEM I were adapted from existing data provided by the cement production company, “Cement Molins Industrial (CMI)”, located in Barcelona. The Ci values of CEM I and CEM II were 0.753 kg CO_2_-_I_ and 0.674 kg CO_2_-_II_, respectively. The supply and transport of raw materials and cement production have been included in the given values. The CO_2_ emissions per kg of FA were considered as 0.023 kg CO_2_-_FA_ (including transportation), a similar value considered by Matos et al. [41]. Natural and recycled aggregates employed in this study were produced within 30 km of Barcelona, having similar environmental costs [2]. Therefore, the aggregates used were not considered in the analysis. The chemical admixtures or amount of electricity employed for concrete production were also not taken into account. They could be valued as having approximately 10% of the total impact of the global warming potential (CO_2_-eq m^−3^) [1], a value similar to that in all of the produced concretes. The eco-efficiency of RCAs owing to the reduction of landfill use and the low consumption of natural resources was not considered either, as there is no significant difference in direct CO_2_ emissions. The use of recycled aggregates significantly reduces the consumption of natural resources [3,54]; however, this was not considered in this work. The absorption of CO_2_ during concrete carbonation was also not considered. Consequently, the Ci results probably underestimate the impact on global warming. However, the obtained data are still helpful in analysing the potential detrimental effect on the environment.

The CO_2_ emissions per m^3^ of concrete are determined by Equation (1).

CO_2_-_CONCRETE_ = CEM (II or I) × CO_2_-_I/II_ + FA × CO_2_-_FA_ (1)

Finally, the concretes’ Ci value was determined by dividing the CO_2_ emission (in kg) per m^3^ of concrete by its unit of performance. Compressive strength was expressed as kg CO_2_·m^−3^ MPa^−1^, carbonation as kg CO_2_ m^−3^ mm^−1^ year^0.5^ and chloride resistance performance as kg CO_2_ m^−3^ C^−1^.

The lower Bi-comp and Ci-comp values obtained guarantee a higher efficiency of required strength in the concrete. Whereas, a higher Bi and Ci for carbonation and chloride resistance guarantee a higher efficiency in achieving required durability values within the concrete. Moreover, only the concrete that achieved the durability property values (carbonation and chloride resistance) defined by the Spanish Structural Code [55] for XC3 and XC4 exposition environment and the ASTM C1202 specifications [53], respectively, whether similar or not too high, could be considered eco-efficient concretes.

**Figure 4:**
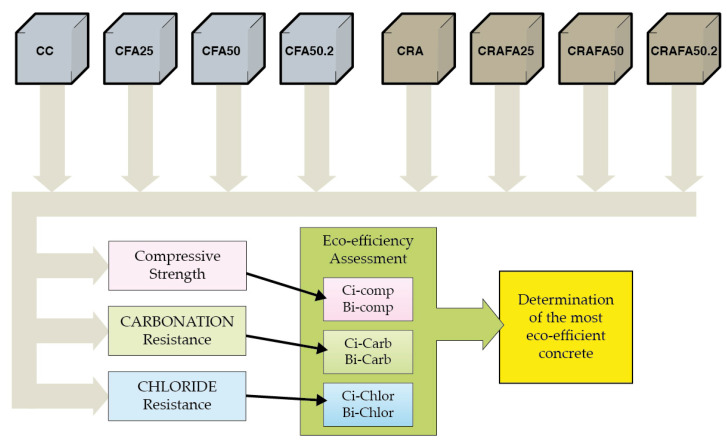
Flowchart of the test procedure carried out.

Figure 4 describes the flowchart of the test procedure and the analysis carried out.

## 3. Results

### 3.1. Compressive Strength

The compressive strength and the standard deviation of all concretes at 7, 28 and 90 days are shown in Table 3. The CC-1 and CC-2 mixtures achieved the highest compressive strength in Series 1 and Series 2, respectively, at tested ages, 7, 28 and 90 days.

**Figure 5:**
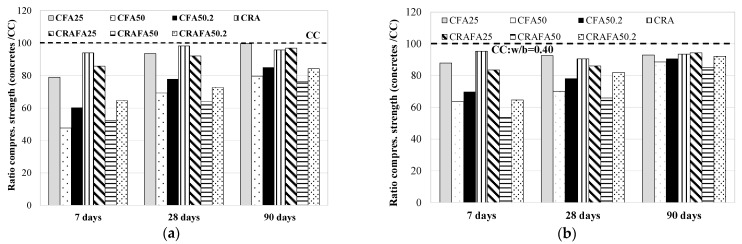
The compressive strength of all concretes with respect to the CC concrete: (**a**) Series 1, (**b**) Series 2.

The concretes produced with FA had a lower initial strength than the CC concretes. In Series 1 (Figure 5a), at 28 and 90 days, CFA25-1 achieved similar strength to the CC-1 concrete. On the contrary, at 28 and 90 days, the CFA25-2 concrete (Series 2, Figure 5b) suffered a reduction of 13% and 7%, respectively, compared to that of the CC-2 concrete. In addition, all the 50% FA concretes tested achieved lower strength values than those of the CC concretes at all the tested ages. Similar results were obtained by Nath and Sarker [56] at 28 and 90 days.

In addition, the CFA50-1 and CFA50-2 concretes with FA replacing CEM II were found to have lower strength values than the CFA50.2-1 and CFA50.2-2 concretes, when FA was the replacement of CEM I. While it was determined that in Series 2, the CFA50.2-2 concrete achieved a <10% reduction of the compressive strength compared to the CC-2 concrete at 90 days, the CFA50.2-1 concretes produced in Series 1 achieved a decrease of 15% with respect to the CC-1 concrete.

According to the results obtained from the concretes produced with RCA, the CRA-1 and CRA-2 concretes achieved compressive strength levels similar to CC-1 and CC-2, respectively, at all ages. While the CRA-1 achieved a maximum reduction of 6% with respect to CC-1 concrete at 7 days, the CRA-2 achieved a maximum reduction of 9.5% with respect to CC-2 at 28 days. In Series 1, FA in the production of RAC led to a lower reduction of the compressive strength compared to that of the FA concretes produced with natural aggregates at 7 days of curing. Probably, this difference was due to a reaction to the FA caused by the presence of a slightly higher amount of portlandite in the concretes produced with uncarbonated RCA (See Figure 2, uncarbonated RCA). In addition, in both series, the CRAFA25 concretes achieved similar (at 28 days) and higher (at 90 days) strength than the CRA concretes. It must be noted that a minute reaction occurred when a high volume of FA was employed in the concrete production [24,57], due to the reduction of the compressive strength value for the 50% FA replacement. Nonetheless, in Series 2, the influence of 50% FA was more prominent. While CRAFA50.2-1 had lower strength than RCA-1 by 26% at 28 days and 12% at 90 days, the CRAFA50.2-2 had 15% and 1.4% lower strength than the RCA-2 concrete.

### 3.2. Durability Properties: Carbonation and Chloride Resistance

Due to the lower compressive strength of the concrete produced with 50% FA in the replacement of CEM II, only the CFA50.2 and CRAFA50.2 concrete mixtures were considered for the durability analysis.

#### 3.2.1. Carbonation Resistance

Table 4 shows the carbonation depth and the compressive strength of the samples submitted to accelerated carbonation at day 0 (after the pre-conditioning) and at 7 and 14 days of the accelerated carbonation process.

The CC and CRA concrete mixtures (produced with 100% of CEM II) achieved the lowest carbonation depth in both Series. In addition, the CC-2 and CRA-2 concretes achieved 0 mm of carbonation after 7 days of the carbonation process (Table 4). Furthermore, it was determined that increased quantities of FA resulted in increased carbonation depth. The carbonation depth increased considerably when 50% FA was employed as this had a lower amount of Ca(OH)_2_ in the cement paste [58], a result which is in line with other research reports [30,38,59].

In Series 1, the concretes produced with RCA suffered lower carbonation depth than those with natural aggregates, probably because RCA concretes had higher amounts of CaO_reacted_ [58]. This lower carbonation depth also occurred in Series 2, where it was found that the carbonation depth of CRAFA25-2 was lower than that of the CFA25-2 concrete. However, when 50% FA was employed, the CFA50.2-040 and CRAFA50.2-2 concretes achieved similar values.

**Figure 6:**
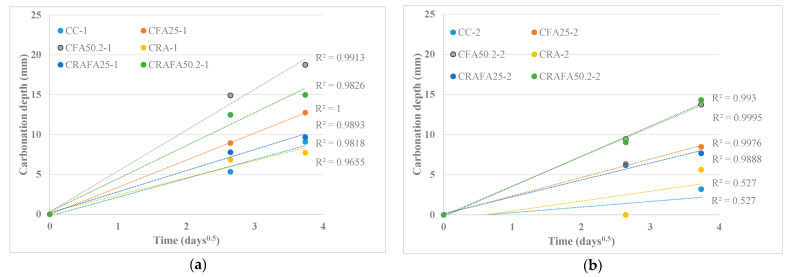
(**a**) Carbonation depth versus time (days^0.5^) at 14 days of exposure to a 20% CO_2_ concentration in Series 1. (**b**) Carbonation depth versus time (days^0.5^) at 14 days of exposure to a 20% CO_2_ concentration in Series 2.

Figure 6a,b show the linear regression of the mean depths as a function of the square root of time (up to 14 days) for the Series 1 and 2 concretes, with the linear regression’s slope representing the carbonation coefficient (*k*_acc_) of each concrete. The *k*_acc_ (Table 4) was determined assuming a steady-state condition (i.e., constant carbonation coefficient) defined by Fick’s first law of diffusion shown in Equation (2).

*X_c_*(*t*) = *k*_acc_·(*t*)^0.5^ (2)

where *X_c_* is the carbonation depth (mm), *k*_acc_ is the carbonation coefficient and *t* is the time (day).

In addition, the natural carbonation coefficient, *k*_nat_, of each concrete was determined (Table 4) assuming the relationship between *k*_acc_ and *k*_nat_, which has been previously defined [60,61] and is shown in Equation (3).

(3) $$\frac{k_{acc}}{k_{nat}}=\frac{\sqrt{\emptyset_{acc}}}{\sqrt{\emptyset_{nat}}}$$

where ∅_acc_ and ∅_nat_ are CO_2_ concentrations in accelerated carbonation (20%) and the natural carbonation process (400 ppm, in Barcelona), respectively.

Figure 6a and Table 4 (see Series 1) show that the CFA50.2-1 concrete achieved the highest *k*_acc_ value (5.6 mm/day^0.5^), followed by the CFA25-1 concrete. The CC-1 concrete achieved the lowest *k*_acc_ (2.3 mm/day^0.5^). However, the use of 50% RCA in concrete production caused a reduction of the *k*_acc_ coefficient compared to those employing the same binder type and natural aggregates. This effect was more evident in concretes produced with FA.

Here, the uncarbonated RCA aggregates were carbonated during their contact with CO_2_, thus causing an increased carbonation resistance. Previous research [16,20] has validated the acceptable behaviour of uncarbonated RCA. Although, up to the current date, the majority of research has concluded that the concrete produced with RCA achieves lower carbonation resistance as a result of the high porosity of the aggregates [17,21,22,30]; moreover, the carbonated or uncarbonated state of RCA could modify this behaviour. The CRA-1, CRAFA25-1 and CRAFA50.2-1 concretes achieved a lower *k*_acc_ coefficient by 8.3%, 22.1% and 18.7% compared to the CC-1, CFA25-1, and CFA50.2-1 concretes, respectively.

In Series 2 (Figure 6b), the CC-2 concrete achieved the lowest *k*_acc_ value, followed by the CRA-2 concrete. The CRA-2 concrete achieved a higher carbonation coefficient, probably because the quality of the new cement paste was superior to that of RCA, thus causing an increase in the carbonation coefficient [22]. The fact that the CC-2 and CRA-2 concretes achieved an *R*-squared value of 0.53, much lower than any other concrete, is probably due to the high CO_2_ concentration resulting in an acceleration of the carbonation process from 7 to 14 days. However, testing the lower CO_2_ concentration would be required to verify the carbonation coefficient value. The *R*-squared value of all the other concrete mixtures was >0.80 (the majority achieving *R* = 0.99), guaranteeing that at 7 and 14 days, the relationship between carbonation depth and time was constant.

**Figure 7:**
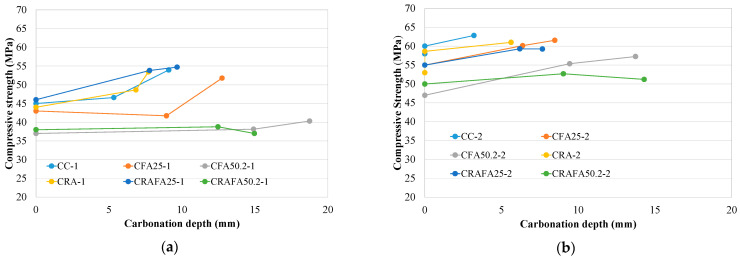
(**a**) Carbonation depth versus compressive strength for the carbonated samples in Series 1. (**b**) Carbonation depth versus compressive strength for the carbonated samples in Series 2.

Figure 7a,b show the relationship between the compressive strength of the carbonated concrete samples from 0 to 14 days versus carbonation depth for the Series 1 and 2 concretes, respectively. The concretes with higher compressive strength achieved lower carbonation depth (Figure 7a), and this conclusion is supported by previous research [21,36,39]. On day 0 of the carbonation process, the compressive strength of the concretes depended only on the type of binder employed in concrete production and not on RCA. Nevertheless, the carbonation depth of concrete produced with RCA was lower than that produced with natural aggregates when the same type of binder was employed. Similar results were obtained by Thomas et al. [16], whose research revealed that the carbonation rate of RAC was slightly lower than for the CC concrete with respect to similar compressive strength concretes.

The results for Series 2 were similar to those for Series 1 (Figure 7b). In general, the concretes with the higher initial compressive strength achieved the lower carbonation depth. However, in Series 2, the influence of RCA on the carbonation depth was less evident. Nevertheless, due to the low water/cement value of those concretes, they achieved an adequate carbonation resistance, as previously demonstrated [36,39].

**Figure 8:**
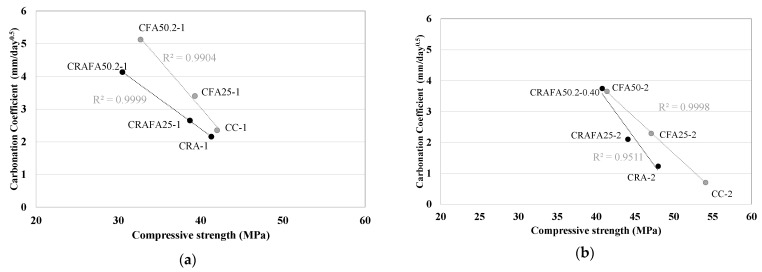
Compressive strength versus carbonation depth for all the concretes. (**a**) Series 1 and (**b**) Series 2. The concretes produced with RCA are represented by the black dots.

Figure 8a,b indicate the carbonation depth versus the compressive strength at 28 days for all concretes (Series 1 and 2). The carbonation depth levels depended on the concrete’s compressive strength at 28 days. All the concretes produced with RCA in Series 1 achieved similar compressive strengths and a lower carbonation depth than those made with natural aggregates. In contrast, in Series 2, the employment of RCA caused a reduction of the compressive strength, but the carbonation coefficient was similar when FA was employed in concrete production.

According to Silva et al. [18], to avoid corrosion of concretes exposed to environmental conditions of the classes XC3 and XC4 (defined in the EN 206-1 specification), the maximum accelerated carbonation coefficient should be 35 mm/year^0.5^ for XC3 and 50 mm/year^0.5^ for XC4 for a service life of 50 years. Consequently, the results obtained in the present paper estimated that the Series 1 CC-1, CRA-1 and CRAFA25-1 concrete mixtures would be adequate for use only in an XC4 environment with the *k*_acc_ values of 45, 41 and 49 mm/year^0.5^, respectively. The XC3 environment, being less restrictive, also allowed a smaller cover width of the concrete structure. In addition, the Series 2 CC-2 and CRA-2 concretes were adequate for employment in both XC3 and XC4 environments, and the CFA25-2 and CRAFA25-2 concretes would also be suitable for use in an XC4 environment.

According to the Spanish Structural Concrete Code [55], the minimum cover in an XC3 environment for a service life of 50 and 100 years should be 20 mm and 30 mm, respectively. In the case of concrete exposed to an XC4 environment for a service life of 50 and 100 years, the minimum cover should be 25 mm and 35 mm, respectively. Therefore, in accordance with the natural carbonation coefficient, *k*_nat_, determined by Equation (3) and described in Table 3, and considering the minimum covers defined by the Structural Code, the Series 1 CC-1, CRA-1 and CRAFA25-1 concretes would be adequate for both the XC3 and XC4 environments. Furthermore, the CFA25-1 concrete could also be used in an XC4 environment for both service life periods. The Series 2 CC-2, CFA25-2, CRA-2 and CRAFA25-2 concrete mixtures would be adequate for an XC3 environment, and the six analysed concretes, including those produced with 50% FA, would be adequate for an XC4 environment for a service life of 50 and 100 years. However, further tests should be carried out to verify those behaviours in an accelerated carbonation process of high CO_2_ concentration.

#### 3.2.2. Chloride Resistance

**Figure 9:**
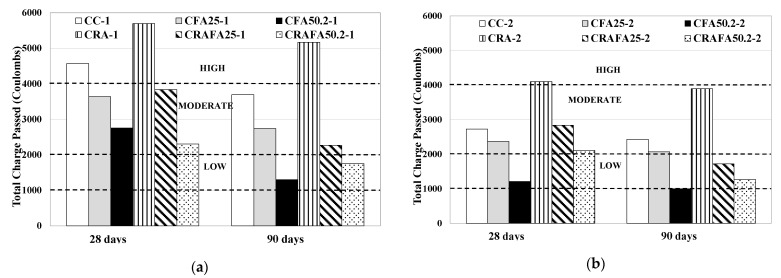
Chloride-ion penetration of concrete mixtures and ASTM corrosion ranges. (**a**) Series 1, (**b**) Series 2 concrete.

Figure 9 shows the chloride penetration resistance at 28 and 90 days for each concrete. They are categorised as low, moderate and high corrosion risk level in accordance with the value of the total passed charge.

Figure 9a describes Series 1 concretes at 28 days. The concretes CC-1 and CRA-1 were classified as high corrosion risk level. All the concretes produced with FA were classified as moderate risk level. The 50% FA concretes (CFA50.2-1 and CRAFA50.2-1) achieved the lowest values of total passed charge. The use of RCA in concrete production was found not to increase the corrosion risk level. Probably, as mentioned by Xiao et al. [20], the use of FA would have resulted in an improvement of the internal pore structure, not only decreasing the porosity of RAC but also improving the interfacial transition zone (ITZ) between the coarse RCA and the new cement paste. This achievement was also described by Sunayana and Barai [31].

At 90 days, the 50% FA concretes (CFA50.2-1 and CRAFA50.2-1) achieved the lowest values, followed by the CRAFA25-1 concrete. The concretes produced using 25% FA and RCA reduced the corrosion risk with respect to concretes produced with FA and natural aggregates. However, the CRA-1 concrete was found to still have a high corrosion risk level after 90 days of curing. While the reduction of the total passed charge from 28 to 90 days for the CFA50.2-1, CFA25-1 and CC-1 concrete mixtures was 53%, 25% and 19%, respectively, the reduction rate of CRAFA50.2-1, CRAFA25-1 and CRA-1 was 24%, 41% and 9%, respectively. CFA50.2-1 achieved the highest decrease, followed by the CRAFA25-1 and CRAFA50.2-1 concretes.

In contrast, the Series 2 concrete samples (Figure 9b) after 28 consecutive days of curing were classified as moderate corrosion risk level, with the exception of the CRA-2 and CFA50.2-2 concretes which were classified as high and low corrosion risk level, respectively. Testing samples at 90 days demonstrated that while the CC-2, CFA25-2 and CRA-2 concretes maintained a moderate corrosion risk level, CFA50.2-2 achieved a negligible corrosion risk level and the CRAFA25-2 and CRAFA50.2-2 concretes achieved low corrosion risk levels. With respect to the decrease of the total passed charge from 28 to 90 days, while CFA50.2-2, CFA25-2 and CC-2 was 18%, 13% and 11%, respectively, the reduction rate of CRAFA50.2-2, CRAFA25-2 and CRA-2 was 40%, 39% and 5%, respectively. The concretes produced with RCA and FA achieved the highest reduction of corrosion risk.

### 3.3. Eco-Efficiency of Concretes

The eco-efficiency of concretes was assessed by measuring the Bi and Ci values, taking into account the mechanical and durability performance. To evaluate the Bi values, 350 kg of binder per m^3^ of concrete was used for all the produced concretes. As for the Ci values, three binder mixtures were considered: 100% CEM II, 75% CEM II plus 25% FA and 50% CEM I plus 50% FA.

#### 3.3.1. Eco-Efficiency for Mechanical Performance

**Figure 10:**
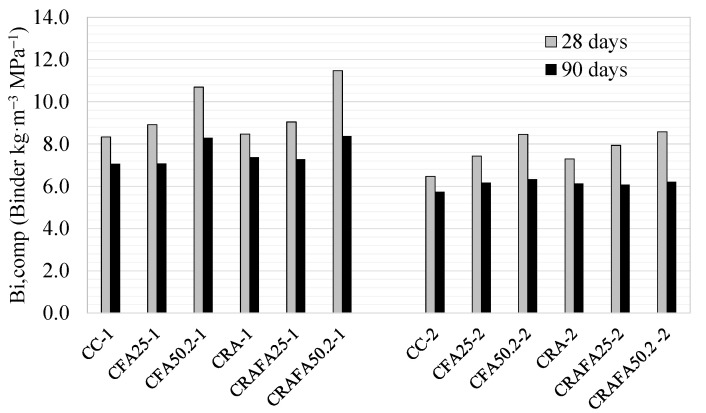
The binder intensity (Bi-comp) for Series 1 and Series 2 concretes at 28 days and 90 days of curing.

The obtained Bi-comp values at 28 and 90 days are illustrated in Figure 10 for all the concretes (the compressive strength values at 28 and 90 days are shown in Table 3). The lower Bi-comp value guarantees a higher efficiency of the concrete for a required strength value.

In Series 1, at 28 days, the CC-1 and CRA-1 concrete mixtures achieved the lowest Bi-comp values of 8.3 kg m^−3^ MPa^−1^ and 8.5 kg m^−3^ MPa^−1^, respectively, whereas, to the contrary, the 50% FA concretes CFA50.2-1 and CRAFA50.2-1 achieved the highest values of 10.7 and 11.5 kg m^−3^ MPa^−1^, respectively. These results are acceptable considering that FA replaced 50% of the cement, and Damineli et al. [4] stated that ready mix concretes < 40 MPa, which utilise the most cement, have a Bi of 10–12 kg m^−3^ MPa^−1^. It was also determined that the effect of RCA on the Bi-comp value was almost negligible in concretes produced with 0% and 25% FA, as RCA did not affect the reduction of compressive strength. After 90 days of curing, the concretes produced with 50% FA achieved the highest reduction of Bi-comp compared to the values at 28 days. The CFA50.2-1 and CRAFA50.2-1 concretes achieved the value of 8.2 kg m^−3^ MPa^−1^. In addition, the CFA25-1 and CRAFA25-1 concretes achieved a similar Bi-comp value to that of the CC or CRA concrete (7.0 kg m^−3^ MPa^−1^).

In Series 2 concretes, the binder achieved higher effectiveness due to the use of a lower water/binder ratio than in Series 1 concretes. This also occurred in Series 1, at 28 days, where it was determined that the CC-2 and CRA-2 concretes achieved the most moderate Bi-comp values (6.5 and 7.3 kg m^−3^ MPa^−1^, respectively). The Bi-comp value was slightly higher when RCA was employed. The concretes CFA50.2-2 and CRAFA50.2-2 achieved the highest values with 8.5 and 8.6 kg m^−3^ MPa^−1^, respectively, because of lower compressive strength. After 90 days of curing, all the Series 2 concretes achieved a similar Bi-comp value, approximately 6 kg m^−3^ MPa^−1^, regardless of the type of binder or aggregates. According to various researchers [4], the Bi-comp values of all concretes were considered acceptable.

**Figure 11:**
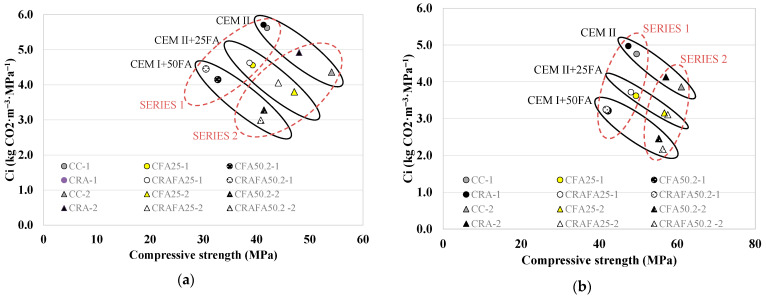
Ci (CO_2_ intensity) versus compressive strength for the concretes at (**a**) 28 days and (**b**) 90 days.

As mentioned above, in order to assess the Ci-comp value it is necessary to determine the kg CO_2_-eq m^−3^ for the produced concretes, different binder types being only considered. A lower Ci-comp value guarantees higher efficiency. Figure 11a,b illustrate the relationship between each concrete’s Ci-comp versus the compressive strength at 28 and 90 days, respectively. The concretes produced with a higher amount of FA and the highest compressive strength [1] were found to achieve the lowest Ci-comp value, and consequently, were considered the most eco-efficient mixtures.

Figure 11a shows that the Series 1 CC-1 and CRA-1 concretes achieved a Ci-comp value of 5 kg CO_2_ m^−3^ MPa^−1^. However, the CFA25-1 and CRAFA25-1 concretes achieved a lower Ci-comp value (4.1 kg CO_2_ m^−3^ MPa^−1^) as they achieved compressive strengths similar to CC-1 and CRA-1 concretes. The concrete produced employing 50% FA achieved the lowest Ci values due to the upper range of the FA’s cement replacement [42]. However, they had the lowest compressive strength. The values in Series 1 were within the range of the average estimated Ci-comp for international values [4]. In Series 2, RCA negatively affected the compressive strength of concrete [9,62], as a consequence of which the CRA-2 concrete achieved the highest Ci value (4.4 kg CO_2_ m^−3^ MPa^−1^). It was determined that the 50% FA concretes were the most efficient concretes. The CRAFA50.2-2 concrete achieved the lowest value with 2.7 kg CO_2_ m^−3^ MPa^−1^ followed by the CFA50.2-2 concrete with the value of 3.0 kg CO_2_ m^−3^ MPa^−1^, representing concretes that were considered very efficient [41].

At 90 days of curing (Figure 9b), the concretes belonging to the same Series achieved similar compressive strength values, with an evident improvement of Ci-comp values in FA-containing concretes [42]. All the concretes achieved a Ci-comp value lower than 5 kg CO_2_ m^−3^ MPa^−1^, which is considered to be efficient [41]. In Series 1, the CRAFA25-1 concrete achieved an adequate relationship between both the Ci-comp and the compressive strength values. In Series 2, the CRAFA50.2-2 concrete achieved a Ci-comp value of 1.9 kg CO_2_ m^−3^ MPa^−1^ with a compressive strength of 56 MPa (7% lower than that of CC-2). The concretes produced with RCA, 25% and 50% of FA, achieved similar values to those with natural aggregates.

#### 3.3.2. Eco-Efficiency for Durability Performance

According to the durability performance, higher values of Bi and Ci define a higher eco-efficiency of the concretes. Table 5 shows the obtained results. With respect to the binder efficiency and CO_2_-eq intensity in carbonation resistance in Series 1, the concrete with the highest efficiency was CRA-1, followed by the CC-1, CRAFA25-1 and CFA25-1 concretes. In Series 2, due to the lack of positive influence of RCA, the CC-2 concrete had the highest efficiency, followed by the CRA-2, CRAFA25-2 and CFA25-2 concretes.

**Figure 12:**
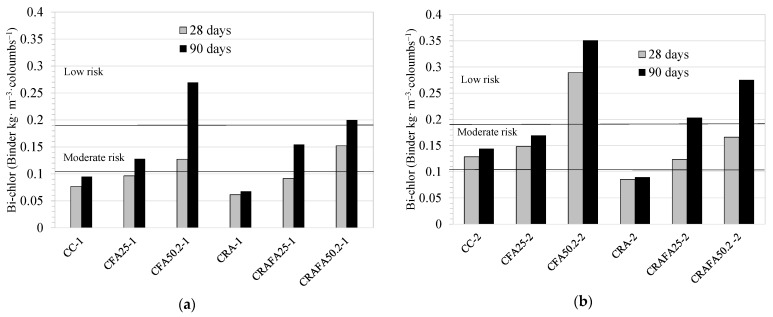
Bi-chlor values for the (**a**) Series 1 and (**b**) Series 2 concretes.

Figure 12a,b show the Bi values for the Series 1 and Series 2 concrete mixtures, respectively, according to chloride resistance (Bi-chlor). The employment of FA improved the binder’s efficiency with respect to chloride resistance. In addition, the use of RCA in concrete did not modify the risk level of chloride attack.

Table 5 shows the Ci-chlor values for the Series 1 and Series 2 concretes at 28 and 90 days. At 28 days, the Series 1 CRAFA50.2-1 concrete achieved the highest eco-efficiency, followed by CC-1, CFA25-1 and CFA50.2-1. At 90 days, although the CFA50.2-1 concrete achieved the highest efficiency, the CRAFA25-1 and CRAFA50.2-1 concretes using RCA also achieved high eco-efficiency. In Series 2, at 28 days, CRA50.2-2 achieved the highest eco-efficiency, followed by the two concretes produced with natural aggregates. At 90 days, the CRAFA25-2 and CRAFA502-2 concretes were also considered to be of high eco-efficiency.

## 4. Discussion

**Figure 13:**
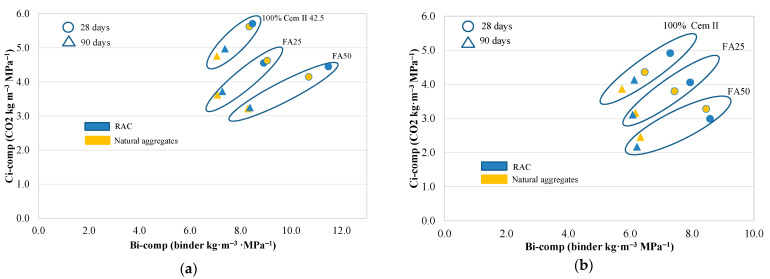
The Bi-comp versus Ci-comp relation for (**a**) Series 1 and (**b**) Series 2.

The concretes produced with the lowest Bi-comp and Ci-comp values were considered the most eco-efficient. Figure 13a,b show the Bi-comp versus Ci-comp values for Series 1 and Series 2 concretes, respectively. In Series 1 (Figure 13a), the concretes produced with 25% FA presented the highest eco-efficiency. The employment of RCA did not decrease the compressive strength of the concrete. Consequently, the CRAFA25-1 was considered the most eco-efficient material. In Series 2 (Figure 13b), at 28 days, the concretes produced with 25% FA (CFA25-2 and CRAFA25-2) were considered the most eco-efficient. However, at 90 days, the CRAFA50.2-2 concrete was the most eco-efficient, followed by CFA50.2-2, CRAFA25-2 and CFA25-2.

To assess the concrete’s eco-efficiency for carbonation resistance, it was considered that the reinforced concrete should be exposed to an XC4 environment for 100-year service life. According to the Spanish Structural Concrete Code [55], the reinforced concrete requires a minimum of 35 mm of cover in that condition, which we considered in this study. Based on those characteristics, the optimum Bi-carb value was determined to be 100 kg·m^−3^·mm^−1^·year^0.5^. Similarly, the optimum Ci-carb values were determined as the maximum kg CO_2_-eq emissions of each concrete made with a specific binder and are presented in Table 6. In addition, Table 6 describes the ratio between the experimental results for the Ci-carb values compared to the optimum Ci-carb value.

The Series 1 CC-1 and CRA-1 concretes achieved the highest ratio values. Consequently, they were considered to be the most durable concretes; however, their CO_2_ emissions proved to be unnecessarily high. The CFA25-1 and CRAFA25-1 concretes were considered the most efficient concretes with values of 1.17 and 1.49, respectively, achieving a value >1, durable enough for this defined environment exposure. In addition, the employment of RCA increased the eco-efficiency using the same amount and type of binder. In contrast, the CFA50.2-1 and CRAFA50.2-1 concretes achieved a ratio value < 1 and these concretes, which cannot be considered durable, do not guarantee the uncarbonation of the cover, thus causing corrosion of the reinforced bars.

All Series 2 concretes were considered durable even though they achieved a ratio value > 1. However, the CFA50.2-2 and CRAFA50.2-2 concretes had the highest eco-efficiency since their ratio value was the closest to 1. Furthermore, due to the reduction of the water/cement ratio, the CC-2 and RCA-2 concretes achieved very high ratio values and excellent durability properties. Still, the kg CO_2_-eq emissions were too high and inadequate for an XC4 environment.

In order to assess the eco-efficiency of the concretes with respect to the chloride ion resistance, it was necessary to achieve a moderate corrosion risk (a maximum charge of 4000 C). Therefore, the optimum Ci-chlor (Table 7) was defined as the maximum kg CO_2_-eq emitted from concretes produced with a specific binder in order to achieve a moderate corrosion risk. The analysis was carried out at 28 days of curing as the values were more critical at this age than at 90 days.

Table 7 shows the optimum Ci-chlor values for each concrete and the ratio of the experimentally obtained values with respect to the optimum value. At 28 days, the Series 1 CC-1 and CRA-1 concretes were not considered durable as the ratio value was <1. The CRAFA50.2-1 achieved the highest value, being the most durable and the most eco-efficient concrete, followed by CFA50.2-1 and CRAFA25-1.

In Series 2, similar to Series 1, the durability and eco-efficiency increased with increasing levels of FA. The CFA50.2-2 achieved the highest value, followed by CRAFA50.2-2. Considering the use of RCA, the CRAFA50.2-1 and the CRAFA50.2-2 concretes had the highest eco-efficiency in Series 1 and Series 2, respectively, making their application possible in this context.

## 5. Conclusions

The following conclusions can be drawn based on the results presented above:

- In concretes produced with a water/cement ratio of 0.50, the use of 50% uncarbonated RCA together with Portland cement did not influence the reduction of compressive strength. In addition, when the concrete was produced with FA, the use of RCA improved the compressive strength of 41.8 MPa compared to concrete employing natural aggregates.
- In concretes produced with a water/cement ratio of 0.40, RCA negatively affected the compressive strength when Portland cement was used for concrete production. However, combining it with FA resulted in the concrete achieving similar compressive strength of 56 MPa to conventional concrete at 90 days.
- Concretes with higher compressive strength achieved higher carbonation resistance. While the use of RCA in concrete production increased the carbonation resistance, it was determined that the employment of FA reduced it. Additionally, the carbonation resistance of concretes produced with FA increased when RCA was used in their fabrication.
- The employment of FA improved the chloride ion penetration resistance of the concrete. In addition, the use of RCA together with FA improved the resistance to chloride ion penetration compared to that of the concretes produced with natural aggregates, even more evidently at 90 days of curing.
- Considering the eco-efficiency analysis:
- Based on the mechanical performance at 28 days, the concretes produced with 25% FA and 50% RCA (CRAFA25) achieved the highest eco-efficiency when an effective water/cement ratio of 0.50 and 0.40 was employed. At 90 days of curing, the CRAFA25-1 and CRAFA50.2-2 concretes achieved the highest eco-efficiency.
- Based on the conclusions of carbonation resistance, the CC and CRA concretes achieved the highest durability and the highest value of eco-efficient values. However, for reinforced concrete exposed to the XC4 environment for 100 years of service life (required 35 mm of cover), the CRAFA25-1 and CRAFA50.2-2 concretes also achieved the minimum durability required according to standards and consequently the highest eco-efficiency.
- Based on the results of minimum chloride resistance where the concretes were required to achieve a moderate corrosion risk: the CRAFA50.2-1 and CRAFA50.2-2 concretes, produced with an effective water/cement ratio of 0.50 and 0.40, respectively, had the highest eco-efficiency. The eco-efficiency of the CRAFA25-1 and CRAFA25-2 concretes was also considered valid.

It can be concluded that the use of 50% uncarbonated RCA as a replacement for natural aggregates combined with FA not only considerably improved the properties of concrete but also their eco-efficiency with respect to concretes produced with natural aggregates. The concrete produced with 50% RCA and 25% FA (CRAFA25) achieved the highest eco-efficiency in accordance with the mechanical performance and carbonation resistance of the concretes. Furthermore, the concrete produced with 50% RCA and 50% FA (CRAFA50, produced with 50% CEM I) had the highest eco-efficiency concerning chloride resistance. All the concretes produced with an effective water/cement ratio of 0.40 were considered durable with respect to carbonation and chloride resistance, independently of the type of binder and aggregates, and met the required standard specifications.

## Figures and Tables

**Table 1:** Composition of binder materials presented as the percentages of the total weight.

	CaO	SiO_2_	Fe_2_O_3_	Al_2_O_3_	MgO	SO_3_	Na_2_O	K_2_O	LOI
CEM II	61.8	18.3	3.3	4.1	1.4	2.9	0.1	0.8	5.1
CEM I	63.5	19.4	3.4	4.2	1.4	3.0	0.12	0.53	3.7
FA	2.3	58.4	7.3	21.6	1.9	0.2	0.9	2.1	3.1

**Table 2:** Mixture proportions of Series 1 and Series 2 concretes. The values are given as weight over volume of concrete production (kg/m^3^) (W: water, Ad: admixture). Ad. was used in % with respect to the cement’s weight

	CEM	FA	W Total	NS	CA1	CA2	RCA	Ad. (%)	Slump Test (cm)
**Series 1**									
CC-1	350	-	192.5	900	275.2	647	-	0.50	20
CFA25-1	262.5	87.5	192.5	867.9	275.2	647	-	0.50	19
CFA50-1	175	175	192.5	835.9	275.2	647	-	0.50	19
CFA50.2-1	175	175	192.5	835.9	275.2	647	-	0.50	19
CRA-1	350	-	213.4	900	137.6	323.5	400	0.50	20
CRAFA25-1	262.5	87.5	213.4	867.9	137.6	323.5	400	0.50	20
CRAFA50-1	175	175	213.5	835.9	137.6	323.5	400	0.50	19
CRAFA50.2-1	175	175	213.5	835.9	137.6	323.5	400	0.50	20
**Series 2**									
CC-2	350	-	157.5	945.3	289.1	679.6	-	1	20
CFA25-2	262.5	87.5	157.5	913.3	289.1	679.6	-	1.1	16
CFA50-2	175	175	157.5	881.2	289.1	679.6	-	1.6	21
CFA50.2-2	175	175	157.5	881.2	289.1	679.6	-	1.6	21
CRA-2	350	-	180.4	945.3	144.5	339.8	425.2	1	18
CRAFA25-2	262.6	87.5	180.4	913.3	144.5	339.8	425.2	1.2	16
CRAFA50-2	175	175	180.5	881.2	144.5	339.8	425.2	1.4	16
CRAFA50.2-2	175	175	180.5	881.2	144.5	339.8	425.2	1.4	16

**Table 3:** Physical properties of concretes produced in Series 1 (with effective w/c of 0.50) and 2 and the compressive strength of concretes at 7, 28 and 90 days. The standard deviation of each average value is given between brackets.

	**Series 1 (Effective w/c 0.50)**			**Series 2 (Effective w/c 0.40)**		
	**Compressive Strength (MPa)**			**Compressive Strength (MPa)**		
	**7 Days**	**28 Days**	**90 Days**	**7 Days**	**28 Days**	**90 Days**
CC	36.7 (0.72)	42.0 (1.4)	49.6 (0.8)	45.1 (0.49)	53.7 (0.41)	61.1 (1.7)
CFA25	29.0 (1.9)	39.3 (1.7)	49.4 (1.7)	39.7 (0.90)	49.7 (1.53)	56.7 (2.15)
CFA50	17.5 (0.8)	29.1 (0.0)	39.5 (1.6)	28.6 (0.05)	37.6 (0.95)	54.1 (0.85)
CFA50.2	22.1 (0.6)	32.7 (0.0)	42.2 (0.8)	31.5 (0.28)	42 (0.17)	55.3 (2.2)
CRA	34.5 (2.4)	41.3 (0.3)	47.5 (1.0)	43.0 (0.95)	48.6 (0.65)	57.1 (1.45)
CRAFA25	31.5 (0.13)	38.7 (0.24)	48.1 (1.1)	37.7 (0.39)	46.2 (2.16)	57.6 (0.6)
CRAFA50	19.2 (0.33)	26.9 (0.98)	37.8 (0.2)	24.2 (0.76)	35.4 (0.58)	51.95 (1.05)
CRAFA50.2	23.7 (0.61)	30.5 (1.9)	41.8 (1.2)	29.1 (1.07)	44.01 (0.76)	56.3 (1.9)

**Table 4:** Carbonation depth and compressive strength after the accelerated carbonation process of all concretes produced in Series 1 and Series 2.

Concrete Type	Carbonation Depth (mm)			*K*_acc_ (mm/day^0.5^)	*K*_nat_ (mm/year^0.5^)	Compressive Strength (MPa)		
	0 Day	7 Day	14 Day			0 Day	7 Day	14 Day
**Series 1 (effective w/c 0.50)**								
CC-1	0	5.3	9.1	2.35	2.08	45.0	46.6	53.9
CFA25-1	0	8.9	12.8	3.4	3.01	43.0	41.7	51.8
CFA50.2-1	0	14.9	18.8	5.1	4.49	37.0	38.1	40.3
CRA-1	0	6.9	7.7	2.16	1.9	44	48.6	53.4
CRAFA25-1	0	7.8	9.7	2.65	2.3	46	53.8	54.7
CRAFA50.2-1	0	12.5	15.0	4.13	3.65	38	38.8	36.8
**Series 2 (effective w/c 0.40)**								
CC-2	0	0.0	3.2	0.702	0.62	58	59.9	61.7
CFA25-2	0	6.34	8.47	2.290	2.03	55	56.7	59.4
CFA50.2-2	0	9.44	13.74	3.651	3.24	47	51.8	58.2
CRA-2	0	0.0	5.63	1.23	1.09	53	55.7	55.1
CRAFA25-2	0	6.19	7.67	2.1025	1.86	55	55.1	55.7
CRAFA50.2-2	0	9.03	14.29	3.7452	3.32	52	50.5	52.5

**Table 5:** Bi and Ci values with respect to carbonation and chloride resistance values of Series 1 and Series 2 concretes. The units of Ci values are expressed as kg CO_2_ m^−3^·mm^−1^·year^0.5^.

	Bi-Carb (Binder kg m^−3^ mm^−1^ year^0.5^)	Ci-Carb (kg CO_2_ m^−3^ mm^−1^ year^0.5^)	Ci-Chlor-28d (kg CO_2_ m^−3^ C^−1^)	Ci-Chlor-90d (kg CO_2_ m^−3^ C^−1^)
**Series 1 (effective w/c 0.50)**				
CC-1	167.85	113.13	0.052	0.064
CFA25-1	116.14	59.38	0.049	0.065
CFA50.2-1	77.81	30.19	0.049	0.105
CRA-1	183.12	123.42	0.041	0.046
CRAFA25-1	149.17	76.27	0.047	0.079
CRAFA50.2-1	95.66	37.12	0.059	0.078
**Series 2 (effect. w/c 0.40)**				
CC-2	564.51	380.48	0.087	0.097
CFA25-2	172.53	88.21	0.076	0.087
CFA50.2-2	108.18	41.98	0.112	0.136
CRA-2	320.52	216.038	0.058	0.061
CRAFA25-2	187.95	96.098	0.063	0.104
CRAFA50.2-2	105.49	36.77	0.058	0.096

**Table 6:** The optimum Ci-carb values of each concrete and the ratios of the obtained results with respect to the optimum results.

	CC	CFA25	CFA50.2	CRA	CRAFA25	CRAFA50.2
Optimum Ci-carb	67.4	51.12	38.8	67.4	51.12	38.8
	**Series 1 (effective w/b = 0.50)**					
Ratio Ci-carb/optimum Ci-carb	1.68	1.16	0.78	1.83	1.49	0.96
	**Series 2 (effect. w/b = 0.40)**					
Ratio Ci-carb/optimum Ci-carb	5.65	1.73	1.08	3.21	1.88	1.05

**Table 7:** The optimum Ci-chlor values of each concrete and the ratios of the obtained results with respect to the optimum results.

	CC	CFA25	CFA50.2	CRA	CRAFA25	CRAFA50.2
Optimum Ci-chlor	0.059	0.045	0.034	0.059	0.045	0.034
	**Series 1 (effective w/b = 0.50)**					
Ratio Ci-chlor/optimum Ci-chlor, 28 days	0.88	1.10	1.45	0.70	1.04	1.74
	**Series 2 (effect. w/b = 0.40)**					
Ratio Ci-chlor/optimum Ci-chlor, 90 days	1.47	1.69	3.30	0.98	1.41	1.90
